# 4-(2-Chloro­ethoxy)phthalonitrile

**DOI:** 10.1107/S1600536808027141

**Published:** 2008-11-13

**Authors:** Xin Huang, Mingfu Li, Xiangmei Lin, Hongjun Chen, Shuifang Zhu

**Affiliations:** a Institute of Animal and Plant Quarantine, Chinese Academy of Inspection and Quarantine, Beijing, 100029, People’s Republic of China

## Abstract

In the title compound, C_10_H_7_ClN_2_O, the O and both C atoms of the chloroethoxy group are disordered over two positions, the occupancy factor of the major disorder component refining to 0.54 (2).

## Related literature

For background to the use of phthalonitriles and phthalocyanines, see: McKeown (1998 ▶); Leznoff & Lever (1989–1996 ▶); Moser & Thomas (1983 ▶). For related structures, see: Nesi *et al.* (1998 ▶); Dinçer *et al.* (2004 ▶); Ocak *et al.* (2004 ▶).
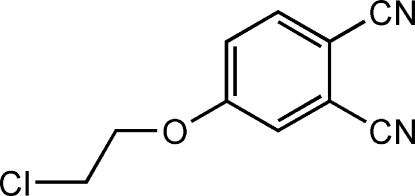


## Experimental

### 

#### Crystal data

C_10_H_7_ClN_2_O
*M* *_r_* = 206.63
Monoclinic, 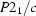

*a* = 4.9021 (8) Å
*b* = 19.014 (3) Å
*c* = 10.640 (3) Å
β = 97.123 (18)°
*V* = 984.1 (3) Å^3^
*Z* = 4
Mo *K*α radiation
μ = 0.35 mm^−1^
*T* = 295 (2) K
0.6 × 0.2 × 0.1 mm

#### Data collection

Bruker P4 diffractometer
Absorption correction: none
2496 measured reflections
1741 independent reflections
890 reflections with *I* > 2σ(*I*)
*R*_int_ = 0.062
3 standard reflections every 97 reflections intensity decay: none

#### Refinement

*R*[*F* ^2^ > 2σ(*F* ^2^)] = 0.065
*wR*(*F* ^2^) = 0.240
*S* = 1.08
1741 reflections
155 parameters
3 restraints
H-atom parameters constrained
Δρ_max_ = 0.27 e Å^−3^
Δρ_min_ = −0.34 e Å^−3^

### 

Data collection: *XSCANS* (Bruker, 1997 ▶); cell refinement: *XSCANS*; data reduction: *XSCANS*; program(s) used to solve structure: *SHELXTL* (Sheldrick, 2008 ▶); program(s) used to refine structure: *SHELXTL*; molecular graphics: *SHELXTL* software used to prepare material for publication: *SHELXTL*.

## Supplementary Material

Crystal structure: contains datablocks I, huangx-4. DOI: 10.1107/S1600536808027141/sj2530sup1.cif

Structure factors: contains datablocks I. DOI: 10.1107/S1600536808027141/sj2530Isup2.hkl

Additional supplementary materials:  crystallographic information; 3D view; checkCIF report

## Figures and Tables

**Fig. 1.**
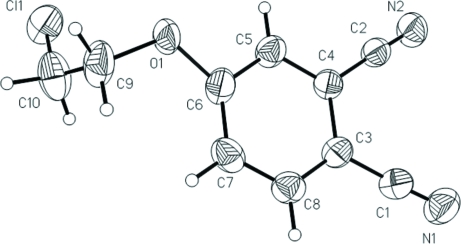
The structure of the title compound, (I), showing 35% probability ellipsoids and the atom numbering scheme. For clarity only atoms of the major disorder component are shown.

**Fig. 2.**
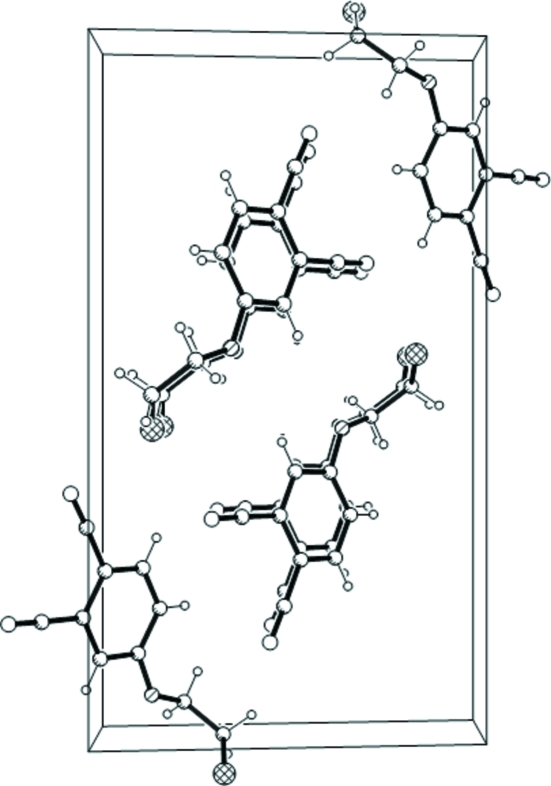
The molecular packing of (I) viewed along the a axis. H atoms and atoms of the minor disorder component have been omitted.

## supplementary crystallographic information

### Comment

Substituted phthalonitriles are generally used for preparing peripherally substituted symmetrical and unsymmetrical phthalocyanine complexes and subphthalocyanines (McKeown, 1998; Leznoff & Lever, 1989–1996). Phthalocyanines were first developed as dyes and pigments (Moser & Thomas, 1983). Over last few years, a great deal of interest has been focused on the synthesis of phthalocyanine derivatives due to their applications in fields, such as chemical sensors, electrochromism, batteries, semiconducting materials, liquid crystals, non-linear optics and photodynamic therapy (PDT) (Leznoff & Lever, 1989–1996). We report here the structure of the title phthalonitrile derivative, (I), (Fig 1).

The title compound, C_10_H_7_ClN_2_O, contains a pathalonitrile ring and 2-chloroethoxy substituent in the 4-position. The oxygen and both carbon atoms of this substituent are disordered over two positions. The occupancy factor of the major disorder component refined to 0.54 (2). The C1≡N1 and C2≡N2 bond distances are both 1.138 (4) °, consistent with N≡C triple-bond character, They are also in good agreement with literature values (Nesi *et al.*, 1998; Dinçer *et al.*, 2004; Ocak *et al.*, 2004).

### Experimental

2-chloroethanol (1.6 g, 20 mmol) and 3-nitrophthalonitrile (1.73 g, 10 mmol) were dissolved in dry dimethylformamide (50 ml). After stirring for 1 h at room temperature, dry fine-powdered potassium carbonate (2.76 g, 20 mmol) was added portionwise over a period of 2 h with stirring. The reaction mixture was stirred for 36 h at room temperature and poured into ice-water (300 g). The product was filtered off and washed with water until the filtrate was neutral. Recrystallization from toluene gave a white product (yield 1.6 g, 77.4%). Single crystals were obtained from ethanol at room temperature by slow evaporation. Spectroscopic analysis: IR (KBr, ν cm^-1^): 2963, 2868, 2237, 2229; MS(ESI, CH_3_OH): m/*z* =207.2 [*M*+H]^+^; Anal. Found: C,58.45; H, 3.72; N, 13.23%. Calcd for C_18_H_16_N_2_O: C, 58.13; H, 3.41; N, 13.56%

### Refinement

The oxygen and both carbon atoms of the chloroethoxy group were disordered over two positions. The occupancy factor of the major disorder component refined to 0.52 (2). All H-atoms were positioned geometrically and refined using a riding model with d(C—H) = 0.93 Å, *U*_iso_=1.2*U*_eq_ (C) for aromatic and 0.96 Å, *U*_iso_ =1.2_eq_ (C) for CH_2_ atoms.

### Crystal data

**Table:**

C_10_H_7_ClN_2_O	*F*_000_ = 424
*M**_r_* = 206.63	*D*_x_ = 1.395 Mg m^−^^3^
Monoclinic, *P*2_1_/*c*	Mo *K*α radiation λ = 0.71073 Å
Hall symbol: -P 2ybc	Cell parameters from 43 reflections
*a* = 4.9021 (8) Å	θ = 4.9–12.4º
*b* = 19.014 (3) Å	µ = 0.35 mm^−^^1^
*c* = 10.640 (3) Å	*T* = 295 (2) K
β = 97.123 (18)º	Prism, colorless
*V* = 984.1 (3) Å^3^	0.6 × 0.2 × 0.1 mm
*Z* = 4	

### Data collection

**Table:**

Bruker P4 diffractometer	*R*_int_ = 0.062
Radiation source: fine-focus sealed tube	θ_max_ = 25.1º
Monochromator: graphite	θ_min_ = 2.1º
*T* = 295(2) K	*h* = −5→1
ω scans	*k* = −1→22
Absorption correction: none	*l* = −12→12
2496 measured reflections	3 standard reflections
1741 independent reflections	every 97 reflections
890 reflections with *I* > 2σ(*I*)	intensity decay: none

### Refinement

**Table:**

Refinement on *F*^2^	Secondary atom site location: difference Fourier map
Least-squares matrix: full	Hydrogen site location: inferred from neighbouring sites
*R*[*F*^2^ > 2σ(*F*^2^)] = 0.065	H-atom parameters constrained
*wR*(*F*^2^) = 0.240	*w* = 1/[σ^2^(*F*_o_^2^) + (0.1087*P*)^2^ + 0.4256*P*] where *P* = (*F*_o_^2^ + 2*F*_c_^2^)/3
*S* = 1.08	(Δ/σ)_max_ < 0.001
1741 reflections	Δρ_max_ = 0.27 e Å^−^^3^
155 parameters	Δρ_min_ = −0.34 e Å^−^^3^
3 restraints	Extinction correction: none
Primary atom site location: structure-invariant direct methods	

### Special details

**Table:**

Geometry. All e.s.d.'s (except the e.s.d. in the dihedral angle between two l.s. planes) are estimated using the full covariance matrix. The cell e.s.d.'s are taken into account individually in the estimation of e.s.d.'s in distances, angles and torsion angles; correlations between e.s.d.'s in cell parameters are only used when they are defined by crystal symmetry. An approximate (isotropic) treatment of cell e.s.d.'s is used for estimating e.s.d.'s involving l.s. planes.
Refinement. Refinement of *F*^2^ against ALL reflections. The weighted *R*-factor *wR* and goodness of fit *S* are based on *F*^2^, conventional *R*-factors *R* are based on *F*, with *F* set to zero for negative *F*^2^. The threshold expression of *F*^2^ > σ(*F*^2^) is used only for calculating *R*-factors(gt) *etc*. and is not relevant to the choice of reflections for refinement. *R*-factors based on *F*^2^ are statistically about twice as large as those based on *F*, and *R*- factors based on ALL data will be even larger.

### Fractional atomic coordinates and isotropic or equivalent isotropic displacement parameters (Å^2^)

**Table:**

	*x*	*y*	*z*	*U*_iso_*/*U*_eq_	Occ. (<1)
Cl1	0.5427 (3)	0.44767 (7)	0.83121 (16)	0.1010 (7)	
N1	0.8645 (10)	0.8557 (2)	0.4503 (5)	0.1033 (15)	
N2	1.0442 (9)	0.6789 (2)	0.3064 (4)	0.0871 (13)	
C1	0.7656 (10)	0.8069 (2)	0.4867 (5)	0.0759 (13)	
C2	0.8952 (9)	0.6778 (2)	0.3795 (5)	0.0689 (12)	
C3	0.6468 (8)	0.7433 (2)	0.5291 (4)	0.0662 (11)	
C4	0.7097 (8)	0.6785 (2)	0.4738 (4)	0.0621 (11)	
C5	0.5977 (8)	0.6167 (2)	0.5135 (4)	0.0707 (12)	
H5A	0.6393	0.5738	0.4785	0.085*	
C6	0.4228 (9)	0.6196 (2)	0.6063 (5)	0.0761 (13)	
C7	0.3573 (9)	0.6827 (3)	0.6596 (4)	0.0755 (13)	
H7A	0.2388	0.6837	0.7214	0.091*	
C8	0.4698 (9)	0.7442 (2)	0.6201 (5)	0.0717 (12)	
H8A	0.4254	0.7869	0.6553	0.086*	
O1	0.297 (3)	0.5510 (8)	0.6161 (17)	0.084 (4)	0.54 (2)
C9	0.134 (4)	0.5396 (9)	0.719 (2)	0.098 (6)	0.54 (2)
H9A	0.0513	0.5837	0.7401	0.118*	0.54 (2)
H9B	−0.0127	0.5068	0.6922	0.118*	0.54 (2)
C10	0.294 (4)	0.5130 (10)	0.8277 (15)	0.101 (6)	0.54 (2)
H10A	0.1641	0.4968	0.8830	0.121*	0.54 (2)
H10B	0.3863	0.5534	0.8694	0.121*	0.54 (2)
O1'	0.352 (4)	0.5616 (10)	0.6669 (13)	0.080 (4)	0.46 (2)
C9'	0.255 (3)	0.5600 (6)	0.7816 (13)	0.059 (4)	0.46 (2)
H9C	0.0815	0.5851	0.7771	0.071*	0.46 (2)
H9D	0.3849	0.5819	0.8458	0.071*	0.46 (2)
C10'	0.2133 (14)	0.4813 (7)	0.8148 (17)	0.070 (4)	0.46 (2)
H10C	0.0970	0.4576	0.7474	0.085*	0.46 (2)
H10D	0.1318	0.4767	0.8930	0.085*	0.46 (2)

### Atomic displacement parameters (Å^2^)

**Table:**

	*U*^11^	*U*^22^	*U*^33^	*U*^12^	*U*^13^	*U*^23^
Cl1	0.0865 (10)	0.0857 (9)	0.1351 (14)	0.0058 (7)	0.0306 (8)	0.0097 (8)
N1	0.114 (4)	0.077 (3)	0.121 (4)	−0.011 (3)	0.024 (3)	0.005 (3)
N2	0.091 (3)	0.088 (3)	0.090 (3)	0.007 (2)	0.041 (2)	0.009 (2)
C1	0.074 (3)	0.070 (3)	0.084 (3)	−0.001 (2)	0.012 (2)	−0.008 (3)
C2	0.075 (3)	0.058 (2)	0.076 (3)	0.001 (2)	0.021 (2)	0.001 (2)
C3	0.064 (2)	0.061 (2)	0.074 (3)	0.0028 (19)	0.008 (2)	0.003 (2)
C4	0.060 (2)	0.062 (2)	0.066 (3)	0.0064 (19)	0.0145 (19)	0.003 (2)
C5	0.073 (3)	0.059 (2)	0.083 (3)	0.008 (2)	0.023 (2)	0.006 (2)
C6	0.079 (3)	0.068 (3)	0.086 (3)	0.008 (2)	0.027 (2)	0.017 (2)
C7	0.072 (3)	0.091 (3)	0.066 (3)	0.019 (2)	0.019 (2)	0.002 (2)
C8	0.070 (3)	0.070 (3)	0.076 (3)	0.009 (2)	0.010 (2)	−0.002 (2)
O1	0.115 (8)	0.067 (6)	0.083 (9)	0.010 (5)	0.062 (7)	0.012 (6)
C9	0.088 (9)	0.096 (9)	0.120 (13)	0.007 (7)	0.053 (9)	0.033 (8)
C10	0.073 (8)	0.141 (15)	0.089 (8)	0.017 (10)	0.015 (7)	0.041 (10)
O1'	0.125 (8)	0.056 (5)	0.066 (8)	−0.004 (5)	0.041 (8)	−0.004 (6)
C9'	0.046 (6)	0.063 (7)	0.071 (8)	−0.009 (5)	0.017 (6)	−0.018 (5)
C10'	0.051 (6)	0.056 (7)	0.109 (10)	−0.020 (5)	0.029 (6)	0.009 (6)

### Geometric parameters (Å, °)

**Table:**

Cl1—C10'	1.726 (5)	C7—H7A	0.9300
Cl1—C10	1.738 (5)	C8—H8A	0.9300
N1—C1	1.137 (6)	O1—C9	1.450 (16)
N2—C2	1.132 (5)	C9—C10	1.41 (3)
C1—C3	1.439 (6)	C9—H9A	0.9700
C2—C4	1.435 (6)	C9—H9B	0.9700
C3—C8	1.378 (6)	C10—H10A	0.9700
C3—C4	1.416 (5)	C10—H10B	0.9700
C4—C5	1.386 (6)	O1'—C9'	1.366 (5)
C5—C6	1.386 (6)	C9'—C10'	1.556 (19)
C5—H5A	0.9300	C9'—H9C	0.9700
C6—O1'	1.344 (17)	C9'—H9D	0.9700
C6—C7	1.382 (6)	C10'—H10C	0.9700
C6—O1	1.452 (15)	C10'—H10D	0.9700
C7—C8	1.381 (6)		
			
N1—C1—C3	177.5 (5)	C10—C9—H9A	109.3
N2—C2—C4	178.3 (5)	O1—C9—H9A	109.3
C8—C3—C4	119.5 (4)	C10—C9—H9B	109.3
C8—C3—C1	121.6 (4)	O1—C9—H9B	109.3
C4—C3—C1	118.9 (4)	H9A—C9—H9B	107.9
C5—C4—C3	119.8 (4)	C9—C10—Cl1	126.4 (13)
C5—C4—C2	121.0 (4)	C9—C10—H10A	105.7
C3—C4—C2	119.2 (4)	Cl1—C10—H10A	105.7
C4—C5—C6	119.1 (4)	C9—C10—H10B	105.7
C4—C5—H5A	120.4	Cl1—C10—H10B	105.7
C6—C5—H5A	120.4	H10A—C10—H10B	106.2
O1'—C6—C7	115.4 (7)	C6—O1'—C9'	125.9 (13)
O1'—C6—C5	121.8 (8)	O1'—C9'—C10'	107.1 (12)
C7—C6—C5	121.5 (4)	O1'—C9'—H9C	110.3
C7—C6—O1	128.9 (7)	C10'—C9'—H9C	110.3
C5—C6—O1	108.7 (7)	O1'—C9'—H9D	110.3
C8—C7—C6	119.2 (4)	C10'—C9'—H9D	110.3
C8—C7—H7A	120.4	H9C—C9'—H9D	108.5
C6—C7—H7A	120.4	C9'—C10'—Cl1	103.4 (9)
C3—C8—C7	120.9 (4)	C9'—C10'—H10C	111.1
C3—C8—H8A	119.6	Cl1—C10'—H10C	111.1
C7—C8—H8A	119.6	C9'—C10'—H10D	111.1
C6—O1—C9	117.8 (11)	Cl1—C10'—H10D	111.1
C10—C9—O1	111.8 (13)	H10C—C10'—H10D	109.0
